# Influence of the size of clonal fragment on the nitrogen turnover processes in a bamboo ecosystem

**DOI:** 10.3389/fpls.2023.1308072

**Published:** 2023-11-23

**Authors:** Zan Zou, Yang Li, Huixing Song

**Affiliations:** ^1^College of Landscape Architecture, Sichuan Agricultural University, Chengdu, China; ^2^Key Laboratory of Ecosystem Network Observation and Modeling, Institute of Geographic Sciences and Natural Resources Research, Chinese Academy of Sciences, Beijing, China

**Keywords:** ramet number, spacer length, nitrogen turnover, heterogeneous light, clonal integration

## Abstract

Different sizes of clonal fragments contain various number of ramets with different spacer lengths, which strongly affects the redistribution of photosynthetic assimilates. Although clonal integration significantly affects rhizosphere processes via microbial enzymes under heterogeneous conditions, the effects of clonal fragment size (ramet number and spacer length) on rhizosphere N turnover processes remain poorly understood. Here, we sampled clonal fragments of *Phyllostachys bissetii* with different ramet numbers and spacer lengths to determine the relative effects of clonal integration and fragment size on rhizosphere processes and resource availability. We found that clonal integration had positive effects on the C and N availability of shaded ramets in clonal fragments with different ramet numbers, owing to the large resource storage in the fragment. However, it only promoted the dissolved organic carbon of the shaded ramets in clonal fragments with different spacer lengths. Results of regression analyses indicated that the response ratios of the soil variables of the shaded ramets first increased when the spacer length was about less than 30 cm and then decreased when the spacer became longer (about >30 cm), suggesting a cost–benefit tradeoff in the fragment. The contribution of the size of clonal fragment to the soil N turnover process was higher than that of clonal integration, whereas its contribution to soil C availability had the opposite effect. These results further revealed the mechanism of the size of clonal fragment in affecting the rhizosphere processes of stressed ramets, which is critical for the adaptation of *P. bissetii* to stressed habitats and further bamboo ecosystem N turnover under climate change.

## 1 Introduction

Clonal plant ramets are characterized by resource translocation between connected ramets through stolons or rhizomes, including photosynthetic products (Wang et al., 2004; Chen J. S. et al., 2015), water (Hu et al., 2015), and organic matter nutrients (Li et al., 2018). Such traits are crucial for clonal plants to adapt to the heterogeneous light environments that are common in nature (Guan et al., 2023). In heterogeneous environments, the uneven distribution of essential resources increases the difficulty in plant absorption (Wang et al., 2017; Chen et al., 2019; Liang et al., 2020). The source–sink gradient in a clonal fragment enables ramets in resource-poor patches to receive resource support from other ramets in resource-rich patches (You et al., 2023; Zhang et al., 2023). Clonal plants always form different sizes of fragments containing multiple ramets with different spacer length. However, the combined effects of fragment size and clonal integration on nitrogen turnover processes in the rhizosphere remains poorly understood.

The size of clonal fragment may affect resource uptake and translocation (Zheng et al., 2023), plasticity (Ganie et al., 2016), and asexual reproduction (Alpert, 1999; Latzel and Klimesova, 2010). Both spacer length and ramet number can affect the size of a clonal fragment. Differences in spacer length may affect the energy required for resource translocation and limit the intensity of clonal integration (Liu et al., 2004; Li et al., 2008; Yu et al., 2020). Previous studies have shown that the benefits of the physiological integration of water in a clonal fragment of *Indocalamus decorus* and *Populus euphratica* decrease when the spacer length increases (Hu et al., 2015; Zhu et al., 2018). In addition, the ramet number can also lead to differences in the sizes of clonal fragments. Each ramet can serve as a source or sink for the entire clonal fragment, where a very complex process of nutrient translocation occurs (Sheng et al., 2007). As the source of nutrients may vary, the output of nutrients that these ramets can provide and the input of resources from connected ramets may also differ owing to the influence of different generations and developmental stages (Wang et al., 2004). Thus, resource redistribution among ramets and physiological integration into clonal fragments are both influenced by the number of ramets (Hutchings and Wijesinghe, 1997; Chen et al., 2010; Zhai et al., 2022). However, to the best of our knowledge, no previous study has tested the relative importance of spacer length and ramet number in clonal integration or rhizosphere processes.

Nitrogen (N) is a limiting resource for clonal plants (Saitoh et al., 2006; Tian et al., 2023), and is regulated by soil microbial community and extracellular enzyme activity (Kuzyakov, 2002; Jones et al., 2004; Sun et al., 2014). Previous studies have shown that clonal integration leads to an increase in soil carbon (C) availability in the rhizosphere of stressed ramets (Lei et al., 2014; Chen J.S. et al., 2015), which could enhance extracellular enzymes and further prime the decomposition of soil organic matter and the transformation of nitrogen (Li et al., 2019). Numerous previous studies have indicated that fragment size can strongly affect the survival, regrowth, and biomass of clonal ramets based on the tradeoff between costs and benefits (Dong et al., 2012; Lin et al., 2012; Huber et al., 2014). Several studies have tried to reveal the influence of the size of clonal fragments on water integration (Hu et al., 2015; Zhu et al., 2018). However, the effect of differences in the sizes of clonal fragments on rhizosphere processes, such as nitrogen turnover, remains largely unknown.

Bamboo, a typical rhizome clonal plant, can undergo clonal integration through connected spacers (Song et al., 2016), resulting in a higher ecological adaptability to heterogeneous light environments (Saitoh et al., 2002; Zheng and Lv, 2023). *Phyllostachys bissetii* is a dominant species in the middle and lower canopy layers of forests and strongly influence ecosystem functions of forest (Kang et al., 2019). In this study, we sampled clonal fragments of *P. bissetii* of different sizes, including different spacer lengths and ramet numbers, to determine the effect of the size of clonal fragment on clonal integration and rhizosphere processes. In this study, we aimed to 1) determine the effects of clonal integration on rhizosphere C and N availability, 2) investigate the effects of the size of clonal fragment on rhizosphere N turnover processes, and 3) estimate the relative contributions of clonal integration and size of clonal fragment to rhizosphere processes. We hypothesize that 1) connected ramets promote rhizosphere processes of shaded ramets through clonal integration, and 2) clonal integration and size of clonal fragment affect rhizosphere processes differently.

## 2 Materials and methods

### 2.1 Experimental design

*P. bissetii* is a perennial and monopodial bamboo species, which is a woody clonal plant that propagates vegetatively by extending its rhizome. *P. bissetii* forms dense rhizome networks in belowground, and new ramets develop from active nodal buds on the rhizome. *P. bissetii* has significant economic value and is one of the main food sources for giant pandas.

**Figure 1:**
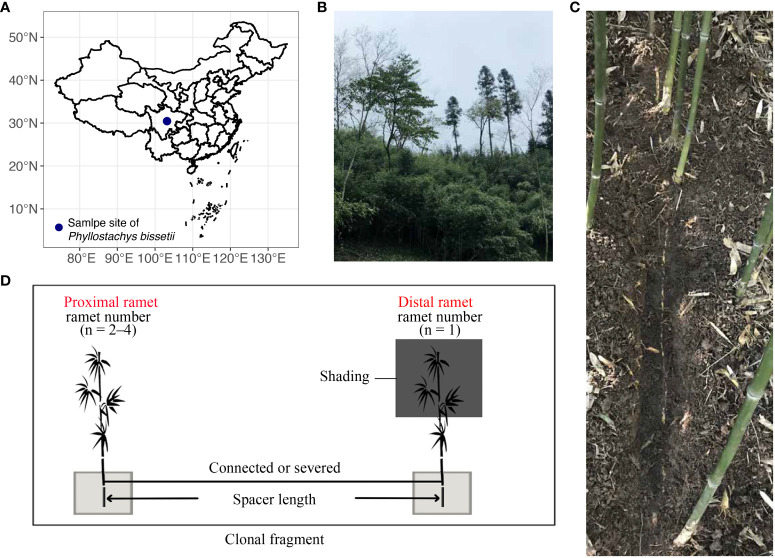
Field design for this study. **(A)** Geographical locations of the experimental areas. **(B)** habitat of *P. bissetii*. **(C)** The rhizome of a clonal fragment of *P. bissetii*. **(D)** The design of the field experiment included clonal fragments with different spacer lengths and ramet numbers. Shading treatment was applied to the distal ramets. The soil blocks were wrapped in a double-layer plastic film.

The *in-situ* field experiment was conducted at Nanbaoshan Town in Sichuan Province, China (103.019° E, 30.45° N, **Figure 1A**), with an elevation of 1217 m. Mean annual precipitation and temperature of this site is 1117.3 mm and 16.3°C, respectively (Li et al., 2019). In 2017, clonal fragments of different sizes were selected at this site, including clonal fragments with various spacer lengths and numbers of ramets (**Figures 1B, C**). Clonal fragments with different spacer lengths contained one exposed ramet and one shaded ramet, whereas clonal fragments with different ramet numbers included to 3–5 ramets. We recorded all spacer lengths between any two connected ramets in all fragments. Distant ramets were identified based on the direction of rhizome growth (Chen B. J. W. et al., 2015; Zou et al., 2018). All distant ramets at the end of each rhizome were shaded using black-shading netting that transmitted only about 20% of the ambient photosynthetically active photon flux density (PPFD). The remaining ramets in the clonal fragments were placed under natural light. Control treatments were established by severing the rhizomes of connecting shaded ramets and others in each fragment (**Figure 1D**). This experiment aimed to explore the effect of clonal integration on the rhizosphere process of shaded ramets. Therefore, only the soil block (50 × 50 × 50 cm^3^) of shaded ramet was wrapped to isolate the ramet from the environment and exclude external influences. The top surface of the block was covered with a mesh to get rid of any potential effects of leaf litter. Double-layered plastic films were used to wrap the surfaces around the block and the bottom. The length of clonal fragments with different spacer lengths ranging from 6 to 40 cm. There were 20 clonal fragments in this spacer length experiment, including 10 connected clonal fragments and 10 severed ones. Each clonal fragment with different ramet numbers was replicated three times, constituting 18 ramet pairs. The control experiment was conducted in the autumn of 2017.

### 2.2 Soil sampling

In June 2018, the rhizosphere soil of shaded ramets was sampled using the shaking root method (Riley and Barber, 1970). Specifically, we removed non-adherent non-rhizosphere soil by gently shaking it off the roots. The soil that strongly adhered to the root was considered the rhizosphere soil and it was gently brushed off with a sterile brush. Plant debris and gravel were removed manually. The samples were then sieved (< 2 mm) and stored at −20°C in the laboratory for chemical analyses.

### 2.3 Rhizosphere soil properties

Total organic carbon (TOC) and total nitrogen (TN) were measured using an element analyzer (Elementar vario MACRO cube, Frankfurt, Germany). Dissolved organic carbon (DOC) and dissolved organic nitrogen (DON) were extracted using KCl solution and then measured using a TOC/TN analyzer (TOC-L analyzer, Shimadzu, Kyoto, Japan). Microbiomass carbon (MBC) and microbiomass nitrogen (MBN) were extracted using the chloroform-fumigation extraction method at atmospheric pressure (CFAP) (Witt et al., 2000; Setia et al., 2012), and their DOC and DON levels with and without CFAP treatment were determined using a TOC/TN analyzer. Soil microbial biomass was measured using the chloroform-fumigation method reported by Vance et al. (1987) and Wu et al. (1990).

The inorganic nitrogen (NH_4_^+^-N and NO_3_^−^-N) content of soil samples (5 g/sample) was extracted using KCl solution and then determined using indophenol-blue colorimetry and dual-wavelength colorimetry (Carter and Gregorich, 2007). Equal amounts of soil samples (5 g/sample) were incubated at 40°C for seven days, and the contents of NH_4_^+^-N and NO_3_^−^-N were measured after incubation. N mineralization (N_min_) and nitrification (N_nitri_) rates were calculated based on the method reported by Zhou et al. (2011).

### 2.4 Soil enzyme activity

The activity of N-acetyl-β-D-glucosaminidase (NAGase) was examined using the method developed by Parham and Deng (2000), where 4-nitrophenyl-N-acetyl-β-D-glucosaminide (ρNP-NAG) was the substrate and the enzyme activity was expressed as μg ρNP g^−1^ soil h^−1^. Urease assay was performed as reported by Kandeler and Gerber (1988) with urea as the substrate and reported in μg NH_4_^+^-N soil h^−1^. The activity of polyphenol oxidase (POXase) was determined using catechol as the substrate (Perucci et al., 2000), with the activity reported in μmol oxidized catechol g^−1^ min^−1^.

### 2.5 Statistical analyses

All statistical analyses were performed using R (v.4.3.1; http://www.r-project.org/), unless otherwise stated. The natural log-transformed response ratio (log-RR) was used to evaluate the effects of clonal integration on measured variables (Hedges et al., 1999).


$$log_{e}RR=\;log_{e}{\bar{X}}_{connected}-log_{e}{\bar{X}}_{severed}$$

where ${\bar{X}}_{connected}$ and ${\bar{X}}_{severed}$ are the mean values of a given variable in the connected and severed shaded ramets, respectively. Random-effect models were used to test whether the effects of clonal integration on measured variables differed from zero with the function “rma” in the package “metafor” (Viechtbauer, 2010). Linear relationships were tested between spacer length and log-RR values of each variable. A linear mixed model was adopted to test the effects of clonal integration and ramet number on the measured variables. The linear mixed model included spacer length as a random effect. The linear mixed models was implemented using the function “lmer” in the package “lme4” (Bates et al., 2015). Finally, variation partitioning was used to test the relative contributions of clonal integration and size of clonal fragment (spacer length and ramet number) on rhizosphere soil carbon related processes, nitrogen pools, and nitrogen processes with the function “varpart” in the package “vegan” (Oksanen et al., 2022).

## 3 Results

### 3.1 Effects of clonal integration on measured variables

**Figure 2:**
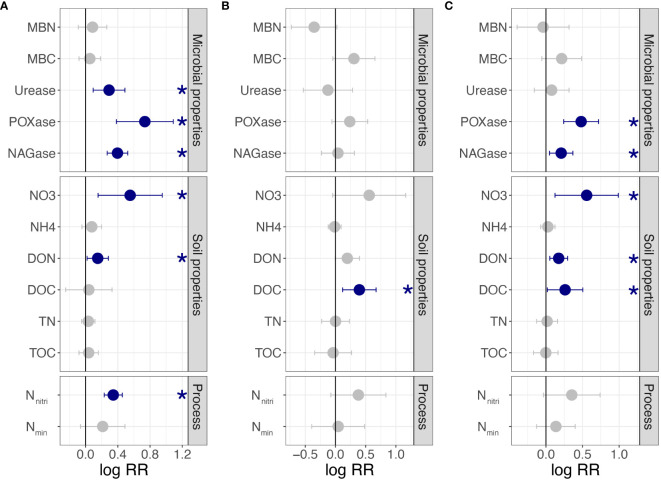
Effects of clonal integration on soil variables in the rhizosphere soil of shaded *P. bissetii* ramets **(A)** from clonal fragments with different ramet numbers, **(B)** from clonal fragments with different spacer lengths, and **(C)** from all clonal fragments. Solid lines indicate the boundary lines of the difference between connected and severed ramets. Blue points indicate the values of the connected treatments are significantly higher than that of the severed treatment. Significance is indicated by *(*p*< 0.05). RR, response ratio; MBN, microbiomass nitrogen; MBC, microbiomass carbon; POXase, polyphenol oxidase; NAGase, N-acetyl-β-D-glucosaminidase; NO_3_, NO_3_^−^-N; NH4, NH_4_^+^-N; DON, dissolved organic nitrogen; DOC, dissolved organic carbon; TN, total nitrogen; TOC, total organic carbon; N_nitri_, N nitrification rate; N_min_, N mineralization rate.

For clonal fragments with different ramet numbers, urease, POXase, NAGase, NO_3_^−^-N, DON, and N_nitri_ were significantly affected by connected or severed rhizomes. All variables of the shaded ramets were significantly higher when the rhizomes were connected (*p<* 0.05; **Figure 2A**). However, MBN, MBC, NH_4_^+^-N, DOC, TN, TOC, and N_min_ were not significantly affected by the connected or severe treatments (*p* > 0.05, **Figure 2A**). For clonal fragments with different spacer lengths, a significant effect was observed only for DOC (*p<* 0.05; **Figure 2B**). By combining all clonal fragments with different ramet numbers and spacer lengths, POXase, NAGase, NO_3_^−^-N, DON, and DOC of connected ramets were significantly higher than those of severed ramets (*p<* 0.05; **Figure 2C**).

### 3.2 Relationships between RR and spacer length

**Figure 3:**
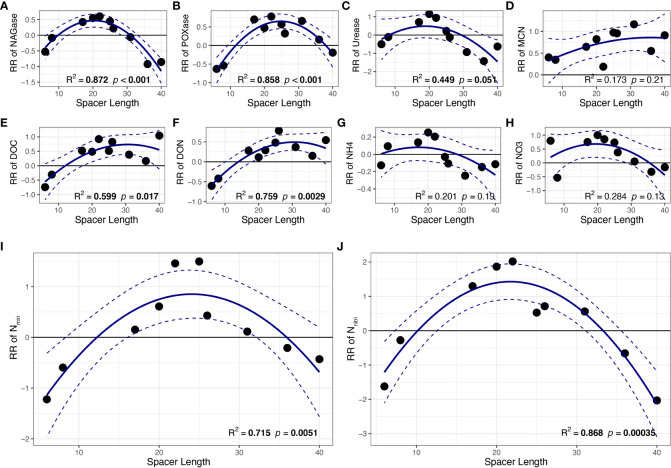
The relationships between spacer length and response ratio of soil **(A)** NAGase, **(B)** POXase, **(C)** Urease, **(D)** MCN, **(E)** DOC, **(F)** DON, **(G)** NH_4_^+^-N, **(H)** NO_3_^−^-N, **(I)** N_min_, **(J)** N_nitri_. Mean regression lines (solid lines) and associated 95% confidence intervals (between two dashed lines) were estimated from the regression analysis with the best goodness-of-fit. Bold values denote the relationship with R^2^ > 0.5 and *p <* 0.05. MCN, microbiomass carbon: nitrogen ratio. All other abbreviations are as in **Figure 2**.

Regression analysis indicated that the response ratios of NAGase, POXase, DOC, DON, N_min_ and N_nitri_ of the shaded ramets were significantly correlated with spacer length (*p<* 0.05; **Figures 3A, B, E, F, I, J**). Results showed that the response ratios of these soil variables first increased and then decreased with the size of clonal fragment, indicating unimodal relationships between all response ratios of these soil variables and spacer length with maximum values. However, no significant differences were observed among the other soil traits, such as urease, MCN, NH_4_^+^-N and NO_3_^−^-N (*p* > 0.05, **Figures 3C, D, G, H**).

### 3.3 Contributions of clonal integration and size of clonal fragment to soil variables

**Figure 4:**
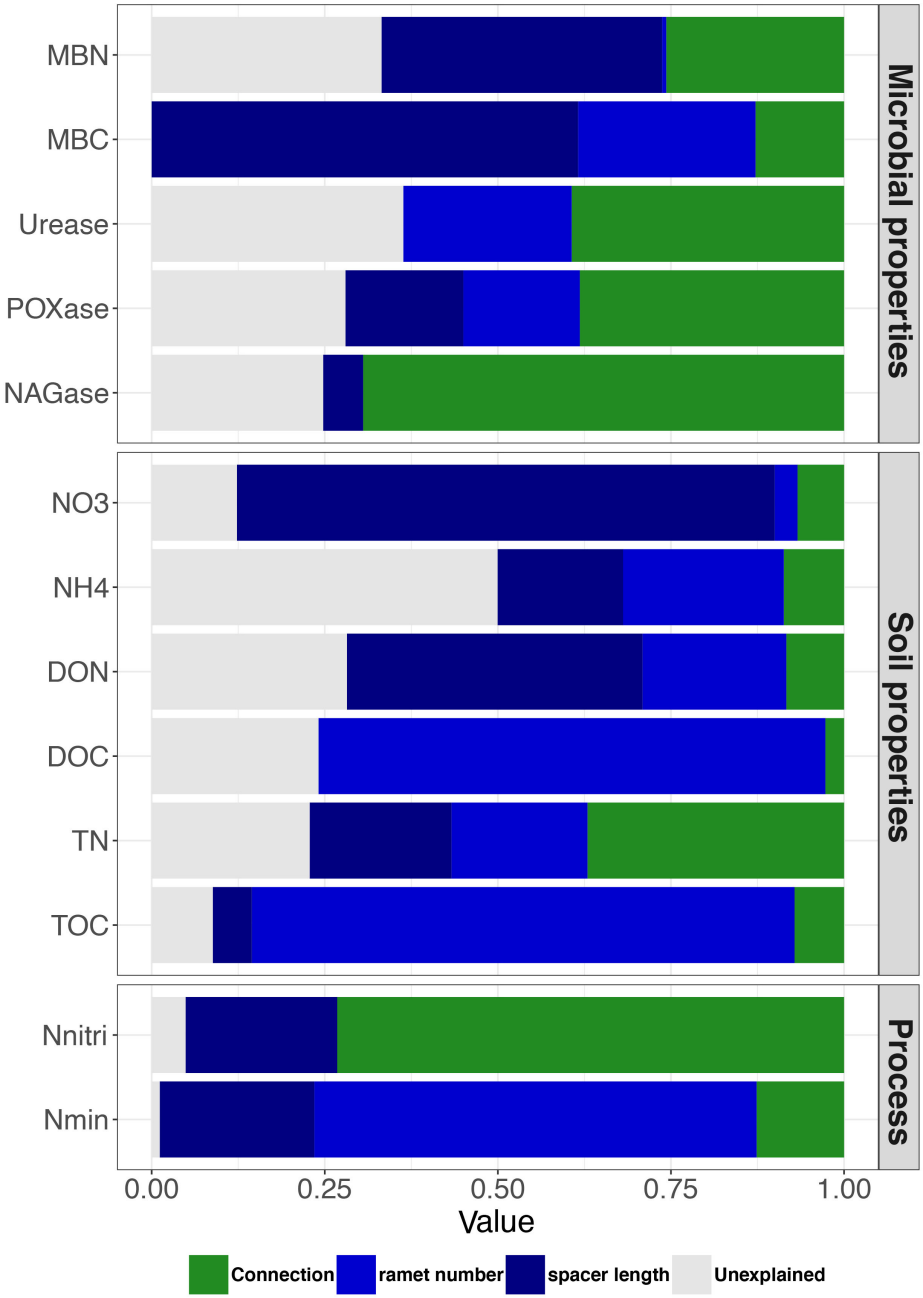
Variation partitioning showing the contribution of clonal integration (connection) and size of clonal fragment (ramet number + spacer length) to soil variables. Green, light blue, dark blue and gray columns represent connection, ramet number, spacer length, and unexplained experiment result, respectively. Abbreviations are as in **Figure 2**.

Based on linear mixed models, we disentangled the relative effects of clonal integration and size of clonal fragment (combining the contributions of ramet number and spacer length) on variations in soil variables. The contribution of the size of clonal fragment to MBN, MBC, NO_3_^−^-N, NH_4_^+^-N, DON, DOC, TN, TOC, and N_min_ was higher than that of clonal integration. Conversely, the contribution of the size of clonal fragment to urease, POXase, NAGase and N_nitri_ expression was lower than that to clonal integration. Furthermore, ramet number mainly contributed to DOC, TOC, and N_min_, whereas spacer length mainly contributed to MBN, MBC, NO_3_^−^-N, and DON (**Figure 4**).

**Figure 5:**
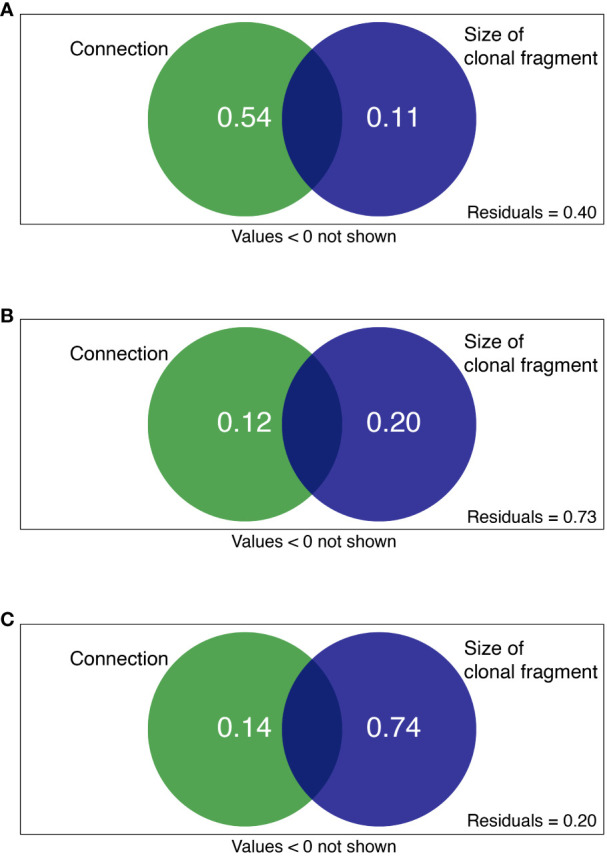
Variation partitioning showing the contribution of clonal integration (connection) and size of clonal fragment (ramet number + spacer length) to soil **(A)** C availability, **(B)** N availability, and **(C)** N turnover process. Green and blue circles represent connection and size of clonal fragment treatments, respectively.

Clonal integration and size of clonal fragments together explained 60, 27, and 80% of the total variation in C availability, N availability, and N turnover processes, respectively (**Figure 5**). For the rhizosphere soil C availability of the shaded *P. bissetii* ramets, the contribution of clonal integration was higher than that of the size of clonal fragment (0.54 > 0.11, **Figure 5A**). However, for the soil N turnover process, the contribution of clonal integration was lower than that of the size of clonal fragment (0.14< 0.74, **Figure 5C**). Similar contributions were observed between clonal integration, size of clonal fragment, and rhizosphere N availability (**Figure 5B**).

## 4 Discussion

We found that clonal integration significantly increased the soil enzyme activity and resource availability in the rhizosphere of shaded ramets, supporting our first hypothesis. Substrates translocate among rhizome-connected ramets in heterogeneous environments, significantly increasing C availability in shaded ramets (Zou et al., 2018). On the one hand, increased C can serve as a convenient substrate for microbes and stimulate microbial activities in the rhizosphere (Kaiser et al., 2010), especially extracellular enzyme activities, which play pivotal roles in soil organic matter degradation and N cycling processes (Hu et al., 2023). However, nitrogen uptake and utilization by shaded ramets may also be enhanced by clonal integration with increased C availability (Li et al., 2018). Thus, clonal integration enables ramets in resource-poor patches to receive support from ramets in resource-rich patches, thereby enhancing the adaptability of the entire clone fragment to heterogeneous habitats (Song et al., 2013; Lu et al., 2020; You et al., 2023).

Our results showed that clonal fragments with different ramet numbers had greater promoting effects on rhizosphere soil properties than clonal fragments with different spacer lengths. This phenomenon can be attributed to the following factors. Each connected ramet serves as a source or sink for an entire clonal fragment (Sheng et al., 2007). Thus, entire clonal fragments with large ramet numbers are more likely to acquire, store, and allocate resources (Dong et al., 2010; Luo and Zhao, 2015), leading to stronger physiological integration (Zhou et al., 2017). This implies that larger clonal fragments have the potential to produce more biomass and exhibited greater competitive ability, especially under low-nutrient conditions (Roiloa and Retuerto, 2005; Zhang et al., 2019). By contrast, a large number of ramets in clonal fragments may also lead to intense intraspecific competition for space and nutrients between sibling ramets (Hellstrom et al., 2006; Zhang et al., 2020). Therefore, the positive effects of clonal fragments with different numbers of ramets are context-dependent and require further attention.

In addition, spacer length influences the effects of clonal integration on the shaded *P. bissetii* ramet. It is noteworthy that these resources are not transmitted indefinitely. According to the results, the response ratio gradually decreased with increasing spacer length in the later stages, indicating a tradeoff strategy between the cost and benefit of clonal integration. This tradeoff is due to the fact that resources translocate to distant ramets in the energy-consumption process. All clonal fragments must adopt a cost–benefit tradeoff strategy when the costs outweigh the benefits of resource translocation (Wang et al., 2014; Zhang et al., 2020). Results showed that this cost–benefit tradeoff strategy occurred when the spacer length was approximately 30 cm (**Figure 3**), which was different from the results for *Populus euphratica* (20–30 m) (Zhu et al., 2018), *Halophila stipulacea* (2.7* cm*), and *Cymodocea nodosa* (81* cm*) (Marba et al., 2002). This may be due to differences in species of clonal plants (D’Hertefeldt et al., 2014; You et al., 2014). Furthermore, to balance the cost and benefit, clonal fragments may even generate new ramets instead of resource translocation (Zhai et al., 2022). Therefore, spacer length strongly affected the clonal integration intensity of *P. bissetii* and had a negative effect over a longer distance.

Clonal integration and fragment size had different effects on rhizosphere processes. The results showed that clonal integration mainly affected the rhizosphere soil enzyme activity related to soil organic matter decomposition (Kaiser et al., 2010; Chen J. S. et al., 2015; Xue et al., 2018). It has been suggested that C assimilates are the major substrates translocated between connected ramets under heterogeneous light conditions (Li et al., 2019). Photosynthetic carbon is an indispensable part of the carbon cycle in the plant–soil system, which is translocated from exposed ramets and compensates for the decreased C inputs to the soil in shaded ramets (Lei et al., 2014; Wang et al., 2019). Thus, clonal integration could supply substrates for microbes and facilitate enzyme activity and further organic matter decomposition in the rhizosphere of the shaded ramets. By contrast, the size of clonal fragment strongly contributed to rhizosphere N turnover. Nitrogen is always heterogeneously distributed in the soil matrix, which increases the difficulty in the accessibility of N to plants. Therefore, clonal fragments may change the placement of ramets, ramet generation, and spacer length to acquire essential nutrients, leading to a strong contribution to rhizosphere N processes (Ma et al., 2023). In addition, the size of clonal fragment also contributes more greatly to the soil variables (such as inorganic N, DOC and DON) than clonal integration. This further implies the potential role of fragment size and structure in adapting to heterogeneous habitats. In total, through resource translocation and regulation of the size of clonal fragment, clonal plants can strongly affect rhizosphere C and N availability and other processes, which eventually enhance the adaptability of the entire clonal fragment to heterogeneous habitats.

## 5 Conclusions

Through field experiments, our study revealed the relative effects of clonal integration and fragment size on rhizosphere processes and resource availability in bamboo ecosystems. We found that clonal integration significantly modified the soil properties of the shaded ramets, whereas the positive effects differed across clonal fragments with different ramet numbers and spacer lengths. Clonal fragments with more ramets could provide a better support to stressed ramets owing to higher resource storage in the fragment. The contribution of spacer length to clonal integration indicated a cost–benefit tradeoff in the fragment. These results advance our knowledge on the mechanism of influence of the size of clonal fragment on rhizosphere processes of stressed ramets, which is critical for the adaptation of *P. bissetii* to stressed habitats and further C cycling through the bamboo ecosystem under climate change.

## Data availability statement

The original contributions presented in the study are included in the article/supplementary material. Further inquiries can be directed to the corresponding authors.

## Author contributions

ZZ: Conceptualization, Data curation, Investigation, Methodology, Software, Writing – original draft. YL: Supervision, Writing – review and editing. HS: Supervision, Writing – review and editing.

## References

[B1] Alpert P. (1999). Effects of clonal integration on plant plasticity in *Fragaria chiloensis* . Plant Ecol. 141, 99–106. doi: 10.1023/A:1009823015170

[B2] Bates D. Machler M. Bolker B. M. Walker S. (2015). Fitting linear mixed-effects models using lme4. J. Stat. Software 67, 1–48. doi: 10.18637/jss.v067.i0

[B3] Carter M. R. Gregorich E. G. (2007). Soil Sampling and Methods of Analysis. 2nd ed (Boca Raton: CRC Press). doi: 10.1201/9781420005271

[B4] Chen B. J. W. Vermeulen P. J. During H. J. Anten N. P. R. (2015). Testing for disconnection and distance effects on physiological self-recognition within clonal fragments of *Potentilla reptans* . Front. Plant Sci. 6. doi: 10.3389/fpls.2015.00215 PMC4387473 25904925

[B5] Chen D. Ali A. Yong X. H. Lin C. G. Niu X. H. Cai A. M.. (2019). A multi-species comparison of selective placement patterns of ramets in invasive alien and native clonal plants to light, soil nutrient and water heterogeneity. Sci. Total Environ. 657, 1568–1577. doi: 10.1016/j.scitotenv.2018.12.099 30677922

[B6] Chen J. S. Lei N. F. Dong M. (2010). Clonal integration improves the tolerance of *Carex praeclara* to sand burial by compensatory response. Acta Oecol. 36, 23–28. doi: 10.1016/j.actao.2009.09.006

[B7] Chen J. S. Li J. Zhang Y. Zong H. Lei N. F. (2015). Clonal integration ameliorates the carbon accumulation capacity of a stoloniferous herb, *Glechoma longituba*, growing in heterogenous light conditions by facilitating nitrogen assimilation in the rhizosphere. Ann. Bot. 115, 127–136. doi: 10.1093/aob/mcu207 25429006 PMC4284106

[B8] D’Hertefeldt T. Enestrom J. M. Pettersson L. B. (2014). Geographic and habitat origin influence biomass production and storage translocation in the clonal plant *Aegopodium podagraria* . PloS One 9, e85407. doi: 10.1371/journal.pone.0085407 24427305 PMC3888427

[B9] Dong B. C. Alpert P. Guo W. Yu F. H. (2012). Effects of fragmentation on the survival and growth of the invasive, clonal plant *Alternanthera philoxeroides* . Biol. Invasions. 14, 1101–1110. doi: 10.1007/s10530-011-0141-5

[B10] Dong B. C. Yu G. L. Guo W. Zhang M. X. Dong M. Yu F. H. (2010). How internode length, position and presence of leaves affect survival and growth of *Alternanthera philoxeroides* after fragmentation? Evol. Ecol. 24, 1447–1461. doi: 10.1007/s10682-010-9390-5

[B11] Ganie A. H. Reshi Z. A. Wafai B. A. Puijalon S. (2016). Clonal growth architecture and spatial dynamics of 10 species of the genus *Potamogeton* across different habitats in Kashmir Valley, India. Hydrobiologia. 767, 289–299. doi: 10.1007/s10750-015-2509-5

[B12] Guan B. Yu J. Wu M. Liu X. Wang X. Yang J.. (2023). Clonal integration promotes the growth of *Phragmites australis* populations in saline wetlands of the Yellow River Delta. Front. Plant Sci. 14. doi: 10.3389/fpls.2023.1162923 PMC10272724 37332707

[B13] Hedges L. V. Gurevitch J. Curtis P. S. (1999). The meta-analysis of response ratios in experimental ecology. Ecology. 80, 1150–1156. doi: 10.1890/0012-9658(1999)080[1150:TMAORR]2.0.CO;2

[B14] Hellstrom K. Kytoviita M. M. Tuomi J. Rautio P. (2006). Plasticity of clonal integration in the perennial herb *Linaria vulgaris* after damage. Funct. Ecol. 20, 413–420. doi: 10.1111/j.1365-2435.2006.01115.x

[B15] Hu J. Chen S. Guo Z. Yang Q. Li Y. (2015). Effects of spacer length on water physiological integration of *Indocalamus decorus* ramets under heterogeneous water supply. Acta Bot. Boreal.-Occid. Sin. 35, 2532–2541. doi: 10.7606/j.issn.1000-4025.2015.12.2532

[B16] Hu M. J. Wang J. L. Lu L. L. Shao P. Zhou Z. Wang D.. (2023). Post-fire soil extracellular enzyme activities in subtropical-warm temperate climate transitional forests. Land Degrad. Dev. 34, 1973–1983. doi: 10.1002/ldr.4582

[B17] Huber H. Visser E. J. W. Clements G. Peters J. L. (2014). Flooding and fragment size interact to determine survival and regrowth after fragmentation in two stoloniferous *Trifolium* species. AoB Plants. 6, plu024. doi: 10.1093/aobpla/plu024 24887003 PMC4062869

[B18] Hutchings M. J. Wijesinghe D. K. (1997). Patchy habitats, division of labour and growth dividends in clonal plants. Trends Ecol. Evol. 12, 390–394. doi: 10.1016/s0169-5347(97)87382-x 21238121

[B19] Jones D. L. Hodge A. Kuzyakov Y. (2004). Plant and mycorrhizal regulation of rhizodeposition. New Phytol. 163, 459–480. doi: 10.1111/j.1469-8137.2004.01130.x 33873745

[B20] Kaiser C. Koranda M. Kitzler B. Fuchslueger L. Schnecker J. Schweiger P.. (2010). Belowground carbon allocation by trees drives seasonal patterns of extracellular enzyme activities by altering microbial community composition in a beech forest soil. New Phytol. 187, 843–858. doi: 10.1111/j.1469-8137.2010.03321.x 20553392 PMC2916209

[B21] Kandeler E. Gerber H. (1988). Short-term assay of soil urease activity using colorimetric determination of ammonium. Biol. Fertil. Soils 6, 68–72. doi: 10.1007/BF00257924

[B22] Kang D. W. Lv J. Li S. Chen X. Wang X. Li J. (2019). Relationship between bamboo growth status and woody plants in a giant panda habitat. Ecol. Indic. 98, 840–843. doi: 10.1016/j.ecolind.2018.12.006

[B23] Kuzyakov Y. (2002). Review: Factors affecting rhizosphere priming effects. J. Plant Nutr. Soil Sci. 165, 382–396. doi: 10.1002/1522-2624(200208)165:4<382::AID-JPLN382>3.0.CO;2-#

[B24] Latzel V. Klimesova J. (2010). Transgenerational plasticity in clonal plants. Evol. Ecol. 24, 1537–1543. doi: 10.1007/s10682-010-9385-2

[B25] Lei N. Li J. Ni S. Chen J. (2014). Effects of clonal integration on microbial community composition and processes in the rhizosphere of the stoloniferous herb *Glechoma longituba* (Nakai) Kuprian. PloS One 9, e108259. doi: 10.1371/journal.pone.0108259 25243590 PMC4171514

[B26] Li Y. Chen J. S. Xue G. Peng. Y. Y. Song. H. X. (2018). Effect of clonal integration on nitrogen cycling in rhizosphere of rhizomatous clonal plant, *Phyllostachys bissetii*, under heterogeneous light. Sci. Total Environ. 628–629, 594–602. doi: 10.1016/j.scitotenv.2018.02.002 29454200

[B27] Li Y. Chen J. S. Xue G. Song H. X. Liu C. H. (2019). Disappearing rhizosphere effect of shaded ramet re-occurs through support of carbon assimilates from unshaded one in a clonal fragment. Rhizosphere. 11, 100166. doi: 10.1016/j.rhisph.2019.100166

[B28] Li Y. H. Liu X. Yue M. (2008). Nutrient contents in *Kingdonia uniflora* ramet and their relations to heterogeneous environment on Taibai Mountains. Chin. J. Appl. Ecol. 19, 1676–1681. doi: 10.13287/j.1001-9332.2008.0298 18975741

[B29] Liang J. F. Yuan W. Y. Gao J. Q. Roiloa S. R. Song M. H. Zhang X. Y.. (2020). Soil resource heterogeneity competitively favors an invasive clonal plant over a native one. Oecologia. 193, 155–165. doi: 10.1007/s00442-020-04660-6 32356013

[B30] Lin H. F. Alpert P. Yu F. H. (2012). Effects of fragment size and water depth on performance of stem fragments of the invasive, amphibious, clonal plant Ipomoea aquatica. Aquat. Bot. 99, 34–40. doi: 10.1016/j.aquabot.2012.01.004

[B31] Liu Q. Li Y. X. Zhong Z. C. (2004). Effects of moisture availability on clonal growth in bamboo *Pleioblastus maculata* . Plant Ecol. 173, 107–113. doi: 10.1023/B:VEGE.0000026334.40661.06

[B32] Lu H. Z. Brooker R. Song L. Liu W. Y. Sack L. Zhang J. L.. (2020). When facilitation meets clonal integration in forest canopies. New Phytol. 225, 135–142. doi: 10.1111/nph.16228 31571219

[B33] Luo W. C. Zhao W. Z. (2015). Burial depth and diameter of the rhizome fragments affect the regenerative capacity of a clonal shrub. Ecol. Complex. 23, 34–40. doi: 10.1016/j.ecocom.2015.05.004

[B34] Ma X. W. Yu W. C. Tao M. Zhang C. Zhang Z. Yu D.. (2023). Clonal integration in *Vallisneria natans* alters growth and the rhizosphere microbial community of neighboring plants under heterogeneous conditions. Plant Soil. 482, 297–311. doi: 10.1007/s11104-022-05690-0

[B35] Marba N. Hemminga M. A. Mateo M. A. Duarte C. M. (2002). Carbon and nitrogen translocation between seagrass ramets. Mar. Ecol. Prog. Ser. 226, 287–300. doi: 10.3354/meps226287

[B36] Oksanen J. Simpson G. L. Blanchet F. G. Kindt R. Legendre P. Minchin P. R.. (2022) Vegan: Community Ecology Package. Available at: https://CRAN.R-project.org/package=vegan.

[B37] Parham J. A. Deng S. P. (2000). Detection, quantification and characterization of β-glucosaminidase activity in soil. Soil Biol. Biochem. 32, 1183–1190. doi: 10.1016/S0038-0717(00)00034-1

[B38] Perucci P. Casucci C. Dumontet S. (2000). An improved method to evaluate the *o*-diphenol oxidase activity of soil. Soil Biol. Biochem. 32, 1927–1933. doi: 10.1016/S0038-0717(00)00168-1

[B39] Riley D. Barber S. A. (1970). Salt accumulation at the soybean (Glycine Max. (L.) Merr.) root-soil interface. Soil Sci. Soc Am. J. 34, 154–155. doi: 10.2136/sssaj1970.03615995003400010042x

[B40] Roiloa S. R. Retuerto R. (2005). Presence of developing ramets of *Fragaria vesca* L. increases photochemical efficiency in parent ramets. Int. J. Plant Sci. 166, 795–803. doi: 10.1086/431804

[B41] Saitoh T. Seiwa K. Nishiwaki A. (2002). Importance of physiological integration of dwarf bamboo to persistence in forest understorey: A field experiment. J. Ecol. 90, 78–85. doi: 10.1046/j.0022-0477.2001.00631.x

[B42] Saitoh T. Seiwa K. Nishiwaki A. (2006). Effects of resource heterogeneity on nitrogen translocation within clonal fragments of Sasa palmata: An isotopic (^15^N) assessment. Ann. Bot. 98, 657–663. doi: 10.1093/aob/mcl147 16845138 PMC3292057

[B43] Setia R. Verma S. L. Marschner P. (2012). Measuring microbial biomass carbon by direct extraction - Comparison with chloroform fumigation-extraction. Eur. J. Soil Biol. 53, 103–106. doi: 10.1016/j.ejsobi.2012.09.005

[B44] Sheng L. Dezhi L. I. Zhiling Z. H. U. Wang X. (2007). Carbon physiological integration in clonal plants and its ecological effects. Chin. J. Appl. Environ. Biol. 13, 888–894. doi: 10.3321/j.issn:1006-687x.2007.06.028

[B45] Song X. Peng C. Zhou G. Gu H. Li Q. Zhang C. (2016). Dynamic allocation and transfer of non-structural carbohydrates, a possible mechanism for the explosive growth of Moso bamboo (*Phyllostachys heterocycla*). Sci. Rep. 6, 25908. doi: 10.1038/srep25908 27181522 PMC4867622

[B46] Song Y. B. Yu F. H. Keser L. H. Dawson W. Fischer M. Dong M.. (2013). United we stand, divided we fall: A meta-analysis of experiments on clonal integration and its relationship to invasiveness. Oecologia. 171, 317–327. doi: 10.1007/s00442-012-2430-9 22915332

[B47] Sun Y. Xu X. Kuzyakov Y. (2014). Mechanisms of rhizosphere priming effects and their ecological significance. Chin. J. Plant Ecol. 38, 62–75. doi: 10.3724/SP.J.1258.2014.00007

[B48] Tian Y. Q. Cui Y. Wen S. H. Li X. Song M. Chen X.. (2023). Clonal integration under heterogeneous water environment increases plant biomass and nitrogen uptake in a temperate steppe. Plant Soil. 491, 145–159. doi: 10.1007/s11104-023-06163-8

[B49] Vance E. D. Brookes P. C. Jenkinson D. S. (1987). An extraction method for measuring soil microbial biomass C. Soil Biol. Biochem. 19, 703–707. doi: 10.1016/0038-0717(87)90052-6

[B50] Viechtbauer W. (2010). Conducting meta-analyses in {R} with the {metafor} package. J. Stat. Software 36, 1–48. doi: 10.18637/jss.v036.i03

[B51] Wang Y. Hong R. Huang D. (2004). The translocation of photosynthate between clonal ramets of *Leymus chinensis* population. Acta Ecol. Sin. 24, 900–907. doi: 10.3321/j.issn:1000-0933.2004.05.006

[B52] Wang Y. J. Müller-Schärer H. Van Kleunen M. Cai A. M. Zhang P. Yan R.. (2017). Invasive alien plants benefit more from clonal integration in heterogeneous environments than natives. New Phytol. 216, 1072–1078. doi: 10.1111/nph.14820 28944478

[B53] Wang C. Xiao R. Cui Y. Ma Z. Guo Y. Wang W.. (2019). Photosynthate-^13^C allocation in the plant-soil system after ^13^C-pulse labeling of Phragmites australis in different salt marshes. Geoderma. 347, 252–261. doi: 10.1016/j.geoderma.2019.03.045

[B54] Wang P. Xu Y. S. Dong B. C. Xue W. Yu F. H. (2014). Effects of clonal fragmentation on intraspecific competition of a stoloniferous floating plant. Plant Biol. 16, 1121–1126. doi: 10.1111/plb.12170 24661501

[B55] Witt C. Gaunt J. L. Galicia C. C. Ottow J. C. G. Neue H. U. (2000). A rapid chloroform-fumigation extraction method for measuring soil microbial biomass carbon and nitrogen in flooded rice soils. Biol. Fertil. Soils. 30, 510–519. doi: 10.1007/s003740050030

[B56] Wu J. Joergensen R. G. Pommerening B. Chaussod R. Brookes P. C. (1990). Measurement of soil microbial biomass C by fumigation-extraction—An automated procedure. Soil Biol. Biochem. 22, 1167–1169. doi: 10.1016/0038-0717(90)90046-3

[B57] Xue G. Li Y. Chen J. Song H. (2018). Effect of clonal integration on soil microbial properties in the rhizosphere of *Phyllostachys bissetii*, subjected to heterogeneous light. Acta Ecol. Sin. 38, 3132–3144. doi: 10.5846/stxb201704030572

[B58] You W. H. Fan S. F. Yu D. Xie D. Liu C. (2014). An invasive clonal plant benefits from clonal integration more than a co-occurring native plant in nutrient-patchy and competitive environments. PloS One 9, e97246. doi: 10.1371/journal.pone.0097246 24816849 PMC4016286

[B59] You W. Li N. N. Zhang J. Song A. Du D. (2023). The plant invader *Alternanthera philoxeroides* benefits from clonal integration more than its native co-genus in response to patch contrast. Plants. 12, 2371. doi: 10.3390/plants12122371 37375996 PMC10302101

[B60] Yu H. W. Wang L. G. Liu C. H. Yu D. Qu J. (2020). Effects of a spatially heterogeneous nutrient distribution on the growth of clonal wetland plants. BMC Ecol. 20, 59. doi: 10.1186/s12898-020-00327-1 33187504 PMC7664100

[B61] Zhai S. S. Qian J. Q. Ma Q. Liu Z. M. Ba C. Q. Xin Z. M.. (2022). Effect of rhizome severing on survival and growth of rhizomatous herb Phragmites communis is regulated by sand burial depth. Plants. 11, 3191. doi: 10.3390/plants11233191 36501231 PMC9736298

[B62] Zhang L. M. Alpert P. Si C. Yu F. H. (2019). Interactive effects of fragment size, nutrients, and interspecific competition on growth of the floating, clonal plant *Salvinia natans* . Aquat. Bot. 153, 81–87. doi: 10.1016/j.aquabot.2018.12.001

[B63] Zhang L. M. Jin Y. Yao S. M. Lei N. F. Chen J. S. Zhang Q.. (2020). Growth and morphological responses of duckweed to clonal fragmentation, nutrient availability, and population density. Front. Plant Sci. 11. doi: 10.3389/fpls.2020.00618 PMC7261891 32523592

[B64] Zhang J. You W. H. Li N. Du D. L. (2023). Invasive clonal plants possess greater capacity for division of labor than natives in high patch contrast environments. Front. Plant Sci. 14. doi: 10.3389/fpls.2023.1210070 PMC10363633 37492774

[B65] Zheng A. Lv J. (2023). Spatial patterns of bamboo expansion across scales: How does Moso bamboo interact with competing trees? Landsc. Ecol. 27, 1–9. doi: 10.1007/s10980-023-01669-z

[B66] Zheng L. L. Yao S. M. Xue W. Yu F. (2023). Small islands of safety promote the performance of a clonal plant in cadmium-contaminated soil. Plant Soil. 489, 453–464. doi: 10.1007/s11104-023-06031-5

[B67] Zhou W. M. Chen H. Zhou L. Lewis B. J. Ye Y. Tian J.. (2011). Effect of freezing-thawing on nitrogen mineralization in vegetation soils of four landscape zones of Changbai Mountain. Ann. For. Sci. 68, 943–951. doi: 10.1007/s13595-011-0100-4

[B68] Zhou J. Li H.-L. Alpert P. Zhang M. X. Yu F. H. (2017). Fragmentation of the invasive, clonal plant *Alternanthera philoxeroides* decreases its growth but not its competitive effect. Flora. 228, 17–23. doi: 10.1016/j.flora.2017.01.007

[B69] Zhu C. G. Li W. H. Chen Y. N. Chen Y. P. (2018). Characteristics of water physiological integration and its ecological significance for *Populus euphratica* young ramets in an extremely drought environment. J. Geophys. Res. Atmos. 123, 5657–5666. doi: 10.1029/2018JD028396

[B70] Zou Z. Chen J. Li Y. Song H. (2018). Effects of transportation direction of photosynthate on soil microbial processes in the rhizosphere of *Phyllostachys bissetii* . Chin. J. Plant Ecol. 42, 863–872. doi: 10.17521/cjpe.2018.0078

